# Kinematic and Morphometric Assessment of Fresh Semen, before, during and after Mating Period in Brahman Bulls

**DOI:** 10.3390/ani14010132

**Published:** 2023-12-29

**Authors:** Ignacio Araya-Zúñiga, Francisco Sevilla, Rafael Molina-Montero, Eduardo R. S. Roldan, Manuel Barrientos-Morales, Miguel A. Silvestre, Anthony Valverde

**Affiliations:** 1 Laboratory of Animal Reproduction, School of Agronomy, Costa Rica Institute of Technology, San Carlos Campus, Alajuela 223-21002, Costa Rica; arayaz.ignacio@gmail.com (I.A.-Z.); fsevilla@estudiantec.cr (F.S.); rafmolina@tec.ac.cr (R.M.-M.); 2 Natural Sciences for Development (DOCINADE), Costa Rica Institute of Technology, San Carlos Campus, Alajuela 223-21002, Costa Rica; 3 Department of Biodiversity and Evolutionary Biology, National Museum of Natural Sciences, Spanish National Research Council (CSIC), 28006 Madrid, Spain; roldane@mncn.csic.es; 4 Laboratory of Reproductive Biology, School of Veterinary Medicine and Animal Science, University of Veracruz, Veracruz CP 91710, Mexico; mbarrientos@uv.mx; 5 Department of Cellular Biology, Functional Biology and Physical Anthropology, Campus Burjassot, University of Valencia, C/Dr Moliner 50, 46100 Burjassot, Spain

**Keywords:** spermatozoa, CASA technology, reproduction, morphology, motility, sperm subpopulation

## Abstract

**Simple Summary:**

Bull fertility greatly impacts the profitability of the cattle production system. Therefore, in vivo semen analysis is an option to predict the potential fertility of the sire. The results showed an effect of the mating period on bull sperm morphometric and displayed spermatozoon with a more linear and faster progressiveness. In addition, we demonstrated an overall decrease in overtime on sperm morphometrical traits of the sperm head and midpiece. Therefore, the results obtained help us understand how the mating period affects a bull’s sperm quality while opening the possibility for future research in other species.

**Abstract:**

The objective of the present study was to determine the effects that the reproductive season has on the motility, kinematics, morphology, and sperm morphometry of Brahman bulls evaluated with a commercial CASA system. The experiment was carried out at the Costa Rica Institute of Technology from March to August 2021. A total of eight Brahman bulls were used. A total of 28 ejaculates were collected in the pre-mating period (PMP), during it (DMP), and after it (AMP) using an electroejaculator. The sperm concentration was measured with the Accuread photometer. The motility was measured using a Spermtrack^®^ counting chamber. The analyses were performed with the CASA-Mot ISAS^®^v1 system. The morphology was analyzed using a microscope with a negative phase contrast objective. Morphometry was evaluated with the CASA-Morph. The sperm concentration did not present differences between the PMP and AMP; however, it was significantly higher than DMP (*p* > 0.05). Regarding the progressiveness variables, linearity on forward progression (LIN), straightness (STR), and wobble (WOB) were higher (*p* < 0.05) DMP. A kinematic principal component analysis grouped all the variables into three factors and an effect on the reproductive period was found (*p* < 0.05) in the parameters of the head and middle part of the sperm, such as width and perimeter, which were greater in the PMP. The length of the sperm head in the PMP and DMP did not show differences; however, both were larger (*p* < 0.05) than AMP. The insertion distance of the middle piece of the sperm was significantly greater than DMP. Finally, the PMP contained cells with a larger insertion angle (*p* < 0.05) than AMP. These findings are important to understand the implications of reproductive status on sperm quality and to consider them in andrological evaluations.

## 1. Introduction

Global beef cattle production faces the challenge of producing more food sustainably with the increasing world population [1]. Successful cattle production systems are linked to the reproductive performance of males and females [2]. How to improve cattle reproductive success could hold an answer to the challenge of food sustainability, increasing the profitability of the system [3], and selecting animals by their potential field fertility [4]. The *Bos indicus* breeds, like Brahman, are the breeds most used in the tropics, and they are adapted to tropical environments [5,6], showing high resistance to high heat stress index and parasites, better feed efficiency on high roughage diets, and efficient thermal exchange [7]. The reproductive performance of Zebu bulls in the harsh conditions of the tropics is considered good compared to European breeds [8]; due to their capacity for thermoregulation, internal temperature requirements can be maintained, to a certain extent, at normal levels for spermatogenesis [9]. In this region, beef cattle production is based on extensive grazing systems and reproduction is mainly based on natural mating with the presence of a single or multiple bulls [9,10,11]. In this context, bull fertility plays a key role in reproductive success [12,13,14,15], and bull selection is very important to improve herd efficiency [10,11]. Infertile or subfertile males are a source of economic loss in beef production systems due to delayed conception, reduced calf weight at weaning, and cow open days [16]. In vitro semen assessment is the best way to predict the potential fertility of a sire [17,18,19]. However, sire sperm quality is multifactorial [20] as it is influenced by breed, health, nutrition, season, and mating system [21].

Sire fertility is mainly associated with the quality of sperm motility and kinematics [22,23]. Furthermore, motility is one of the most used parameters in the world to evaluate semen quality. Notwithstanding, it is not the most determining characteristic when it comes to discriminating a bull as a potential breeder [24]. Sperm kinematics are evaluated with a computer-assisted sperm analysis (CASA) system by capturing a sequence of images of each sperm cell. Then, the system uses algorithms to trace the trajectory of the cells, thus describing their movement [25]. There are a number of external factors that can affect the assessment of motility and kinematics of sperm cells during an automated evaluation [26]. Sperm motility and kinematic patterns can be affected by the type and depth of the counting chamber [25,26,27], temperature [28], extender [28,29,30], frame rate [25,26,27,28,29,30,31], and the dilution used [32]. 

The main objective of the sperm morphometric evaluation is to assess the general structure of the cell [33,34], and it can be useful in predicting the fertility that a male could potentially have [35,36,37]. The morphometry of the cells could be influenced by deficiencies in the normal development of the sperm during spermiogenesis and their maturation along the epididymis [34]. The morphometric assessment can analyze parameters associated with the sperm head, acrosome, and midpiece, and it can be also used to identify morphological abnormalities. However, to identify morphological abnormalities, subjective human intervention is still required [25]. For the analysis in CASA-Morph, the fixation and staining of the spermatozoa are required in such a way that it is easier to capture cell images and identify the possible defects in the acrosome, head, and intermediate piece [38,39].

In species with seasonal reproduction such as deer [40], ram [41], and camel [42], the effect of mating season has been reported. In mammals, seasonal reproduction may be affected by environmental conditions like temperature and photoperiod [42,43]. However, cattle in the tropics are not considered to have a seasonal reproductive pattern because the gonads are functional throughout the year [44]. Some reports highlight that, during the reproductive season, the ejaculates of males present a wide variability, mainly related to sperm velocity patterns in the ejaculate [45,46]. Mating season is a stressful period for males, and it is common for males to lose body weight and condition [47]. However, males must be able to maintain adequate sperm parameters to preserve their potential fertility throughout the mating season. Furthermore, some authors point out that it is important to analyze the semen quality of bulls that are in natural mating at least once a year [48]. Reports in yearling bulls demonstrated that the evaluation of seminal quality during a mating season is a suitable criterion for bull selection [47] because it has been reported that the service capacity of a sire can also be conditioned by a hereditary factor from the father, which is correlated with fertility [49]. In addition, it has to be borne in mind that the selection made in Zebu breeds by the breeding soundness evaluation methodology has not been consistent in comparison to European breeds, and the measured parameters are not sufficient to really be able to diagnose the potential fertility of the sire [50]. Therefore, semen analysis becomes more relevant regardless of the reproduction program used on the farm, whether it is artificial insemination (AI) or natural or controlled mating [8]. In consequence, the aim of the present study was to determine the effect of the mating period on Brahman bull sperm parameters such as motility, kinematics, morphology, and morphometrics using a commercial CASA system.

## 2. Materials and Methods

### 2.1. Ethics

The experiment was conducted at a bovine cattle farm “Finca La Vega”, property of the Costa Rica Institute of Technology, from March to August 2021. The farm is located in the north of Costa Rica (La Vega, 10°25′20.98″ N; 84°31′17.57″ W, Alajuela, Costa Rica, Central America). The study was managed following the laws and regulations for conducting experiments on live animals in Costa Rica. This study was performed following ethical principles and with the approval of the Committee of Centro de Investigación y Desarrollo en Agricultura Sostenible para el Trópico Húmedo at the Costa Rica Institute of Technology (CIDASTH-ITCR), according to Section 01/2019, article 1.0, DAGSC-074-2019. The study was carried out in compliance with ARRIVE guidelines (https://arriveguidelines.org/; accessed on 25 January 2021).

### 2.2. Animals and Mating Period

A total of eight mature bulls of the Brahman breed were used. At the beginning of the experiment, the average age of the males was 57.12 ± 14.50 months. Prior to the start of the experiment, all the bulls had successfully passed a standard breeding soundness evaluation. The bulls were randomized and divided into pairs. Each pair (4 pairs) had one distinct through an 84-day mating season (late May to mid-August). Ejaculates were collected from each pair prior to, during, and after the mating period. Pre-mating period samples (PMP) were collected one to two days before each pair of bulls started mating. Samples corresponding to the post-mating period were obtained 21.88 ± 9.43 days after finishing the period (after the mating period; AMP). For the collection of samples during the mating period (DMP), each pair was in natural mating for at least fourteen days and a maximum of twenty-one days in a breeding herd of 180 cows, with a collection frequency of every seven days. However, due to handling and health issues, one of the animals had to be removed from the study because it did not complete the minimum number of ejaculations per individual. The scrotal circumference (SC) of the bulls was measured prior to each semen collection.

### 2.3. Semen Collection

A total of 28 ejaculates were collected by electroejaculation [51,52]. An automatic program of electrical stimulus was delivered using a 75 mm diameter transrectal probe (Pulsator V, Lane Manufacturing, Denver, CO, USA) with a preprogrammed cycle, alternating stimuli of increasing intensity until ejaculation. Each animal was subjected to up to two cycles of 36 increasingly strong electrical stimuli, each cycle consisting of, first, six stimuli of 1–2 V, nine of 3–5 V, twelve of 6 V, and nine of 7 V. Each stimulus lasted 3.5 s, during which time rectal massage was performed with the probe. Stimulation was halted the moment that ejaculation occurred or when one cycle had been completed without ejaculation according to Toledano-Díaz et al. [53]. If the bull did not ejaculate on the first collection attempt, the animal was allowed to rest for 5 to 8 min before a second attempt was made. Semen samples were collected in sterile 15 mL Falcon tubes; they were then diluted 1:1 (vol/vol) in one step with a commercial extender (Bioxcell^®^; IMV, L’Aigle, France) and prewarmed at 37 °C to avoid temperature shock in Eppendorf^®^ tubes (Sigma-Aldrich, St. Louis, MO, USA). The concentration of each ejaculate was estimated with the Accuread bovine photometer (IMV, L’Aigle, France).

### 2.4. Sperm Kinematics and Motility

To analyze motility, Spermtrack^®^ reusable counting chambers (Proiser R+D, Paterna, Spain) were used after being prewarmed to 37 °C. After thorough mixing of the diluted semen samples, 2.7 µL of diluted semen was placed in the counting chamber. Analyses were conducted with the CASA-Mot system ISAS^®^v1 (Integrated Semen Analysis System, Proiser R+D, Paterna, Spain) fitted with a video camera (Proiser 782 M, Proiser R+D) using a frame rate of 40 frames per second (fps) and a final resolution of 768 × 576 pixels. The camera was attached to a microscope UB203 (UOP/Proiser R+D) with a 1× eyepiece and a 10× negative phase contrast objective (AN 0.25), and an integrated heated stage was maintained at 37.0 ± 0.5 °C. Sperm motility and kinematics were quantified capturing a least seven microscope fields with a minimum of 600 sperm cells per field. The variables in the motility analysis were the percentages of total motility (TM) and progressive motility (PM). The kinematic variables assessed were the rectilinear velocity (VSL), curvilinear velocity (VCL), and average path velocity (VAP) expressed in μm/s. The crossover frequency (BCF) in Hz and the lateral displacement of the head (ALH) in μm were also obtained, in addition to linearity (LIN = VSL/VCL·100), straightness (STR = VSL/VAP × 100), and wobble (WOB = VAP/VCL × 100).

### 2.5. Morphology

Samples were prepared for each bull ejaculate. For each sample, 10 μL of previously diluted and mixed semen was placed on a slide, smeared, and covered with a coverslip, and the slide was introduced into the Trumorph^®^ (Proiser R+D, SL, Paterna, Spain), which subjected the sample to a thermal pulse at 65 °C and a constant force on the coverslip of 20 kiloponds (kp), as previously described by Soler et al. [54]. Once the sample preparation process was completed, the samples were analyzed in a UB203 microscope (UOP/Proiser R+D) with a 1× eyepiece and a 40× negative phase contrast objective to evaluate sperm cells according to their morphology. Two hundred cells per sample were counted and the percentages of normal and abnormal cells were obtained. 

### 2.6. Morphometry

Two subsamples of 10 µL were taken from each ejaculate, placed on a prewarmed slide, smeared, and allowed to air dry. Samples were stained with the Diff-Quick^®^ commercial kit (Medion Diagnostics, Düdingen, Switzerland), according to the manufacturer’s instructions. After placing a coverslip on the stained samples, images were captured under the microscope using a CASA-Morph ISAS^®^v1 system (Proiser R+D). For this analysis, the heads of 120 spermatozoa were randomly captured in different fields, rejecting only those that overlapped with background particles or other cells that interfered with subsequent image processing. Initial erroneous definitions of the sperm head limit and midpiece were corrected by varying the analysis factor of the system. When it was not possible to obtain a correct limit, the sperm was eliminated from the analysis. Five head size measures were taken by the system: length (L, µm), width (W, µm), area (A, µm^2^), perimeter (P, µm), and percentage of head occupied by the acrosome. Four head shape variables were calculated: ellipticity (L/W), rugosity (4πA/P^2^), elongation ((L − W)/(L + W)), and regularity (πLW/4A). Four measurements of the sperm midpiece were quantified: length (µm), area (µm^2^), insertion distance (µm), and insertion angle (°).

### 2.7. Statistical Analysis

The data obtained from the CASA-mot analysis of all sperm motility, kinematics, and morphometric variables were assessed for homoscedasticity using Levene’s tests. A normal probability plot was used to assess the normal distribution. ANOVA was further applied to evaluate statistical differences between treatments for the mating period. Pairwise comparisons between the mating period means were performed using the Tukey–Kramer test. 

#### Multivariate Procedures

Multivariate analyses were performed using principal factor analysis (PFA) to identify sperm clusters and to derive a small number of linear combinations from this subset of sperm kinematics. All values for kinematics variables were standardized to avoid any scaling effects. Prior commonalities for this analysis were estimated from the maximum absolute correlation coefficient between each variable and any other. The number of principal factors (PFs) to be extracted was determined by selecting only those with an eigenvalue >1 (Kaiser criterion). The measure of dataset adequacy for factor extraction was also determined by the KMO (Kaiser–Meyer–Olkin) statistic. The varimax method with Kaiser normalization was used as a rotation method. A two-step cluster procedure with the sperm-derived indices obtained after the PCA was performed. All sperm kinematic variables within each ejaculate were clustered according to velocity, linearity, and oscillation parameters using a non-hierarchical K-means clustering procedure (K-means model and Euclidean distance). ANOVA was further applied to evaluate statistical differences between clusters for all kinematics variables. The threshold for significance was defined as *p* < 0.05. A pairwise comparison between cluster means was performed via the Tukey–Kramer test. The results are presented as mean ± standard deviation of the mean. All data were analyzed using the IBM SPSS statistical program, version 23.0, for Windows (SPSS Inc., Chicago, IL, USA).

## 3. Results

The characteristics of bull ejaculates in relation to the mating period (PMP, DMP, or AMP) are summarized in Table 1. The comparison of the seminal parameters of males prior to, during, and after the mating period did not show an effect of mating activity (*p* > 0.05) on percentages of TM and PM. Sperm concentration did not show differences between the PMP and AMP (*p* > 0.05); however, both were significantly higher than DMP. The bull scrotal circumference did not significantly vary depending on mating PMP, DMP, and AMP. A significant decrease in volume and sperm concentration was observed during the mating period (*p* < 0.05). There were no differences (*p* > 0.05) in percentages of normal sperm morphology and total abnormal sperm.

Sperm kinematics was strongly affected by mating periods (Table 2; *p* < 0.05). Velocities were higher when bulls left the breeding herd (5.21–9.94% and 29.64–33.88%) than in AMP and PMP, respectively. Trajectory variables such as LIN, STR, and WOB, were higher during the mating period (2.44–8.62%) for the PMP and (0.42–3.80%) for AMP. Lateral displacement of the head (ALH) and BCF were higher (*p* < 0.05) after the mating period compared to prior and during mating. 

The effects of different times during the mating period on the sperm head and midpiece parameters were found (*p* < 0.05). Values for the sperm head parameters, such as width, area, and perimeter, were higher in the PMP than DMP and AMP. The sperm head length did not show differences between the PMP and DMP; however, both were larger at these times (*p* < 0.05) than AMP. The sperm form also showed differences (*p* <0.05) and they were higher in the PMP and DMP than AMP: ellipticity (1.876 ± 0.004 and 1.880 ± 0.003) and elongation (0.303 ± 0.001 and 0.304 ± 0.001). Sperm regularity (0.897 ± 0.003) DMP was, in fact, higher (*p* < 0.05) than in the PMP and AMP. Nevertheless, the rugosity of the sperm cell showed significant differences DMP and AMP, with a higher mean AMP (0.757 ± 0.001). 

Regarding sperm midpiece variables, width did not present differences between the PMP and DMP; however, both were higher (*p* < 0.05) than AMP. Sperm with a larger midpiece area were observed AMP (10.87 ± 0.06 µm^2^). The sperm midpiece distance of insertion was significantly higher DMP (0.317 ± 0.003µm). Finally, the PMP (7.92 ± 0.16°) contained cells with higher (*p* < 0.05) insertion angles than DMP (6.82 ± 0.11°) and AMP (5.82 ± 0.16°) (Table 3). 

Principal component analysis grouped all kinematics variables from the PMP, DMP, and AMP into three PC factors. PMP-PC1 factors were associated positively with all trajectory relations (LIN, STR, and WOB) and VCL. PMP-PC2 was related to all velocity parameters (VCL, VSL, and VAP) and ALH. PMP-PC3 was associated with BCF. DMP-PC1 was associated with the same factors as PMP-PC2, while DMP-PC2 was associated positively with sperm linearity index (LIN), straightness (STR), and wobble (WOB). The total variance explained was 89.26%, 90.84%, and 90.72% for PMP, DMP, and AMP, respectively (Table 4).

Sperm subpopulations presented differences depending on the time of mating period (Table 5; *p* < 0.05). PMP-SP3 was the subpopulation that exhibited the highest VCL, VSL, and VAP (154.89 ± 0.69 µm/s, 130.47 ± 0.56 µm/s, and 133.25 ± 0.56 µm/s, respectively). Likewise, PMP-SP3 also included sperm with the highest LIN, STR, and WOB (83.90 ± 0.26%, 95.51 ± 0.20%, and 86.04 ± 0.23%, respectively). More oscillatory movement was seen in PMP-SP1, with higher ALH and BCF (3.08 ± 0.02 µm, 20.25 ± 0.09 Hz). For DMS, the ejaculate subpopulation with the highest velocities (VCL, VSL, and VAP) was DMP-SP3. The DMP-SP2 subpopulation had a higher LIN, STR, and WOB (89.71 ± 0.18%, 95.85 ± 0.14%, and 92.16 ± 0.16%, respectively) than DMP-SP1, DMP-SP3, and DMP-SP4. More undulatory spermatozoa were represented in DMP-SP3, with the highest ALH (4.11 ± 0.01 µm) and BCF (19.00 ± 0.08 Hz). The AMP subpopulations with the highest speeds were AMP-SP4 (VCL, 222.59 ± 1.03 µm/s) and AMP-SP1 (VSL, 153.55 ± 0.65 µm/s and VAP, 165.16 ± 0.65 µm/s). The subpopulation with the highest LIN (84.22 ± 0.26%) and WOB (90.89 ± 0.22%) was AMP-SP1, whereas AMP-SP2 showed higher values for STR (96.25 ± 0.20%). Finally, more undulatory spermatozoa in AMP were grouped in SP4, with higher (*p* < 0.05) ALH (4.20 ± 0.02 µm) and BCF (22.58 ± 0.13 Hz).

## 4. Discussion

Regardless of the type of reproductive strategy used on the farm, whether it is natural mating or artificial insemination, the bull used must have a scrotal circumference higher than thirty-two centimeters to produce large amounts of sperm with normal morphology [16]. Approximately sixty days elapsed between the first and last semen collection, which roughly coincides with the duration of the spermatogenesis process, and our results showed that the morphology of the spermatozoa was not affected during the different times of the mating period (*p* > 0.05). The lack of differences between the ejaculates obtained before and after the mating period suggests that, even though the animals experienced constant sexual activity, sperm morphology was not affected. In dairy cattle, the breeding soundness evaluation methodology has been standardized regarding variables related to bull semen quality before and after the mating season [55]. It has been reported that variations in mean values for sperm motility and morphology do not differ much before and after the mating season, which does coincide with the results observed in this study. However, for this kind of analysis, a standard methodology, which is replicated in the same way in laboratories that analyze semen quality, has not been defined; thus, variability between studies may exist [56]. 

Regarding sperm concentration, studies on *B. taurus* crossbreeds aged between one and two years suggest that animals used during a 28-day breeding season tend to experience a decrease in sperm concentration and an increase in seminal plasma interleukin-8 levels at the end of the mating period [57]. In our work, the bulls were in breeding pastures, in pairs, for a period of twenty-one days, and the mating season extended for approximately eighty days. It has been reported that the potential fertility of a male is dependent on the interaction of multiple factors such as management, environment, and the individual’s own physical and reproductive conditions [47]. Moreover, when analyzing the data before and during the mating period, a similar effect to that described by Snider et al. [57] can be observed, as sperm concentration decreases significantly during the mating period (*p* < 0.05). These authors pointed out that the decline in sperm concentration is of great importance because males showing this effect may potentially be unable to fertilize with females in high male-to-female ratios during the mating season, affecting the pregnancy rate of the productive system.

Motility is one of the most relevant parameters when measuring the potential of sperm to fertilize the egg [15]. The kinematic variables in our study showed a significant increase (*p* < 0.05) over time, which could be attributed to the process of spermatogenesis. This suggests that in the ejaculates collected during the pre-mating period, sperm cells were aged and very close to being reabsorbed, while upon reaching the mating season, on average, these cells were at their peak, approximately 60 days into spermatogenesis. However, some studies in yearling bulls mention that animals may exhibit satisfactory fertility prior to the start of the breeding season, but this condition can be affected once it concludes. In other words, during the mating season, the animal undergoes physical and reproductive changes related to stress conditions generated by reproductive activity. This should take into account the physiology, the environment, and the injuries the animal may suffer, which can limit its fertility [47]. However, our data do not reflect the same behavior. The spermatozoa of domestic animals have a linear movement, which is an adaptation that allows them to avoid the obstacles that arise to reach the ampulla, the site where fertilization takes place [30], although it should be taken into account that species may differ in their pattern of movement [58,59]. Also, it is believed that the adverse conditions within the uterus and oviduct are essential for selecting the most suitable sperm for fertilization [60]. Reports on stallions did not register differences in sperm TM during breeding and resting seasons [61], thus agreeing with the results obtained in this study. Several authors describe a positive effect of collection frequency in bull sperm TM [4], but, in our study, during the mating period, in which there is a higher ejaculation frequency, the opposite effect was observed, with bulls showing the lowest TM, which can be related to a high renewal rate and no adequate maturation of spermatozoa. Likewise, bulls that are not sexually active tend to have reductions in sperm motility [9], although in our study, the effect was different because TM and PM were higher in the PMP and AMP. In any case, these differences were not significant. The results of multivariate analysis revealed three PCs for the prior, during, and after mating sperm kinetics variables. From these PCs, a total of four sperm subpopulations were identified and were consistent with other studies conducted in bulls [62,63]. There are no studies on kinetics subpopulations during a mating period, with prior analyses related to types of semen diluent used [28,29,30,31,32,33,34,35,36,37,38,39,40,41,42,43,44,45,46,47,48,49,50,51,52,53,54,55,56,57,58,59,60,61,62,63], multivariate analysis, sperm velocity and linearity [62], membrane integrity [22], frames per second used [31], and the overall dataset [64]. Our results indicate, in general, that subpopulations with faster and straighter movement were SP3 in PMS, SP3 in DMS, and SP1 in AMS. The results from other studies suggest that bulls whose spermatozoa exhibit more progressive movement and are straighter tend to have higher fertility in vivo [12,23,30,62].

The importance of morphometric analysis of spermatozoa lies in the prediction that can be made about the potential fertility of a male in a productive system [35,36,37]. The results of our morphometric assessments showed a wide variability prior to, during, and after the mating period. Effects on spermatogenesis can give rise to variations in the size of the spermatozoa and could explain the results observed for sperm head and midpiece size and shape variables that were low after mating; it could be specially related with frequency of collection/ejaculation [65] and a competition effect between males [66,67]. Moreover, Kondracki et al. [68] reported in bulls that sperm head size increases as the sperm concentration of the ejaculate decreases. Our results did not show an effect on sperm head size; rather, this effect was observed on the shape of the spermatozoa, which became more elongated, elliptical, and regular during the mating period at concentrations close to 700 million per ml, and it can be also related with the motility of the spermatozoa. The size and the shape of the sperm can affect the kinematic parameters [46,69,70]. The shape of the cell is important because it can reduce fluid resistance to spermatozoa movement [29]. During mating, the sperm cell forms were more elongated, elliptical, and regular, and as our results suggest, this had a positive influence on the linearity and the straightness of the movement. Other authors reported that more elongated spermatozoa tend to present a faster and more linear movement [71], which is in agreement with what was observed in this study during the mating period. Other studies pointed out that the progressiveness of the movement is what dictates the potential fertility of a sire [68]. On the other hand, it may be that the differences in the results for sperm morphometry observed in this study with respect to other studies are largely due to the type of fixation, staining technique used [34,37,38,39], as well as software and settings used [72]. It is also worth considering that the technique of fixation and staining can reduce the size of the head traits [36].

Regarding the variables of the sperm midpiece, there is scanty literature for *Bos indicus* breeds since most studies only focus on the length of the midpiece or on the global analysis of the head. However, some references in dairy bulls [72], vicuna [73], and deer [74] report values for width, area, angle, and insertion distance variables of the midpiece. The relevance of evaluating the midpiece parameters lies in the fact that they present wide variability, which is due to the fact that they are dependent on factors such as age, breed, and the sire [75]. Our results indicated that the status of the bull during the mating period also affects these parameters. The sperm midpiece contains the mitochondria which, through the process of oxidative phosphorylation, provides energy to sperm [76,77]. A hypothesis that is proposed suggests that cells whose intermediate piece tends to be larger present greater energy production in birds [78]; however, this premise does not imply greater speed of movement since it has been shown that the size of the midpiece has a negative correlation with sperm speed in mammals [79]. Furthermore, the size of the midpiece has great relevance to explain the evolution of the species’ spermatozoa, mainly associated with a selection pressure as a consequence of sperm competition [76,78,80].

We should also bear in mind that, during the mating period, the bulls sampled may have mated previously, which could directly affect the parameters of both the motility and kinematic patterns (general means and subpopulation structure), as well as the morphology and morphometry of the spermatozoa. The effect of the sire on the characteristics of the semen should also be also taken into account [29] by not only considering an individual effect, but also the differences that the ejaculates of the same sire can present over time [12,20,41]. In this work, we expanded the perspective on how ejaculate characteristics behave throughout the entire mating season, considering traits before, during, and after the mating period, as well as some features associated with bull semen quality and potential fertility. To the best of our knowledge, similar studies that also consider the behavior of bulls during mating have not been reported. Likewise, our results are based on a wide range of sperm characteristics objectively obtained through CASA technology and by considering variables that provide a more comprehensive description of sperm shape, size, and kinematics. Thus, this work opens a new avenue of research for future studies and emphasizes the importance of evaluating sires from the beginning to the end of the mating season.

## 5. Conclusions

An important effect of the mating period was observed on sperm morphometric and kinematic parameters for Brahman bulls. During the pre-mating period and after the mating period, ejaculate samples presented a higher sperm cell concentration and slightly higher sperm motility (total and progressive). The spermatozoa during the mating period showed a more linear, faster, and straighter movement. Overall, the morphometrical traits of the sperm head and midpiece decreased over time. These findings could be important to help us better understand the impact of mating activity over sperm quality and its value as a condition in breeding soundness evaluation programs. 

## Figures and Tables

**Table 1:** Effect of different times during mating period on seminal characteristics (mean ± SEM) of ejaculates from Brahman bulls.

Variable	Times of Mating Periods		
	PMP	DMP	AMP
SC (cm)	41.21 ± 1.04	41.84 ± 0.74	42.15 ± 1.12
Ejaculate volume (mL)	6.70 ± 1.49 ^a^	2.75 ± 0.97 ^b^	7.79 ± 1.26 ^a^
Concentration (×10^9^ sperm/mL)	1.41 ± 0.25 ^a^	0.66 ± 0.16 ^b^	1.44 ± 0.21 ^a^
TM (%)	75.68 ± 4.13	69.85 ± 2.92	75.82 ± 2.13
PM (%)	65.83 ± 4.13	63.92 ± 2.92	67.90 ± 4.13
Normal sperm (%)	88.81 ± 2.13	89.76 ± 1.51	87.60 ± 2.13
Abnormal sperm (%)	11.19 ± 2.13	10.24 ± 1.51	12.40 ± 2.13

PMP: pre-mating period, DMP: during mating period, AMP: after mating period, SEM: standard error of mean, SC: scrotal circumference, TM: total motility, PM: progressive motility. ^a,b^ Different letters within rows indicate differences in seminal characteristics between different times during mating period. *p* < 0.05.

**Table 2:** Effect of mating period on motility and kinematic sperm variables (mean ± SEM) of ejaculates from Brahman bulls.

Variable	Times of Mating Periods		
	PMP	DMP	AMP
VCL (µm/s)	120.87 ± 0.59 ^c^	141.87 ± 0.50 ^b^	155.97 ± 0.69 ^a^
VSL (µm/s)	86.05 ± 0.52 ^c^	107.71 ± 0.44 ^b^	113.32 ± 0.61 ^a^
VAP (µm/s)	91.98 ± 0.50 ^c^	114.37 ± 0.42 ^b^	123.14 ± 0.58 ^a^
LIN (%)	68.69 ± 0.22 ^c^	74.61 ± 0.18 ^a^	71.88 ± 0.25 ^b^
STR (%)	88.46 ± 0.17 ^b^	90.62 ± 0.14 ^a^	89.03 ± 0.19 ^b^
WOB (%)	74.69 ± 0.16 ^c^	79.98 ± 0.13 ^a^	78.65 ± 0.19 ^b^
ALH (µm)	2.61 ± 0.01 ^b^	2.57 ± 0.01 ^c^	2.68 ± 0.01 ^a^
BCF (Hz)	13.95 ± 0.06 ^c^	15.08 ± 0.05 ^b^	16.49 ± 0.07 ^a^

PMP: pre-mating period, DMP: during mating period, AMP: after mating period, SEM: standard error of mean, VCL: curvilinear velocity, VSL: straight-line velocity, VAP: average path velocity, LIN: linearity of forward progression, STR: straightness, WOB: wobble, ALH: amplitude of lateral head displacement, BCF: beat-cross frequency. ^a–c^ Different letters within rows indicate differences in seminal characteristics between different times during mating period. *p* < 0.05.

**Table 3:** Effect of mating period on morphometry variables of sperm head and midpiece (mean ± SEM) of ejaculates from Brahman bulls.

Variable	Times of Mating Period		
	PMP	DMP	AMP
Sperm head			
Length (µm)	9.41 ± 0.02 ^a^	9.38 ± 0.01 ^a^	9.14 ± 0.02 ^b^
Width (µm)	5.03 ± 0.01 ^a^	5.00 ± 0.01 ^b^	4.95 ± 0.01 ^c^
Area (µm^2^)	41.62 ± 0.08 ^a^	41.07 ± 0.06 ^b^	39.91 ± 0.08 ^c^
Perimeter (µm)	26.29 ± 0.03 ^a^	26.17 ± 0.02 ^b^	25.74 ± 0.03 ^c^
Acrosome (%)	59.86 ± 0.11 ^b^	60.58 ± 0.08 ^a^	59.92 ± 0.11 ^b^
Ellipticity	1.876 ± 0.004 ^a^	1.880 ± 0.003 ^a^	1.851 ± 0.004 ^b^
Rugosity	0.757 ± 0.001 ^ab^	0.754 ± 0.001 ^b^	0.757 ± 0.001 ^a^
Elongation	0.303 ± 0.001 ^a^	0.304 ± 0.001 ^a^	0.297 ± 0.001 ^b^
Regularity	0.894 ± 0.001 ^b^	0.897 ± 0.001 ^a^	0.891 ± 0.001 ^b^
Sperm midpiece			
Width (µm)	1.92 ± 0.01 ^a^	1.91 ± 0.01 ^a^	1.87 ± 0.01 ^b^
Area (µm^2^)	10.69 ± 0.06 ^ab^	10.65 ± 0.04 ^b^	10.87 ± 0.06 ^a^
Insertion distance (µm)	0.307 ± 0.005 ^ab^	0.317 ± 0.003 ^a^	0.303 ± 0.005 ^b^
Insertion angle (°)	7.92 ± 0.16 ^a^	6.82 ± 0.11 ^b^	5.82 ± 0.16 ^c^

PMP: pre-mating period, DMP: during mating period, AMP: after mating period, SEM: standard error of the mean, Length: L, Width: W, Area: A, Perimeter: P, Ellipticity: L/W, Rugosity: 4πA/P2, Elongation: (L − W)/(L + W), Regularity: πLW/4A. ^a–c^ Different letters within rows indicate differences in seminal characteristics between different times during mating period. *p* < 0.05.

**Table 4:** Eigenvectors of principal components (PCs) for kinematic variables of ejaculates according to the different times of the mating period.

Variable	Times of Mating Period								
	PMP			DMP			AMP		
	PC1	PC2	PC3	PC1	PC2	PC3	PC1	PC2	PC3
VCL		0.935		0.966				0.961	
VSL	0.652	0.682		0.834				0.771	
VAP		0.799		0.928				0.900	
LIN	0.963				0.983		0.976		
STR	0.680				0.769		0.720		
WOB	0.930				0.885		0.873		
ALH		0.826		0.765				0.698	
BCF			0.859			0.875			0.854
Var Exp	54.39	25.45	9.43	49.39	30.23	11.20	47.66	29.60	13.46

PMP: pre-mating period, DMP: during mating period, AMP: after mating period, VCL: curvilinear velocity, VSL: straight-line velocity, VAP: average path velocity, LIN: linearity of forward progression, STR: straightness, WOB: wobble, ALH: amplitude of lateral head displacement, BCF: beat-cross frequency, Var Exp: variance explained in each PC. Total variance explained: PMS = 89.26%, DMS = 90.84%, AMS = 90.72%. The table shows the most important variables in each PC. Only eigenvectors > 0.6 are presented.

**Table 5:** Kinematic values (mean ± SEM) of the four sperm subpopulations (SPs) according to the different times during mating period.

Variable	VCL (µm/s)	VSL (µm/s)	VAP (µm/s)	LIN (%)	STR (%)	WOB (%)	ALH (µm)	BCF (Hz)
PMP								
SP1	148.22 ± 0.80 ^b^	96.75 ± 0.65 ^b^	100.10 ± 0.65 ^b^	64.90 ± 0.31 ^c^	94.42 ± 0.23 ^b^	67.15 ± 0.27 ^c^	3.08 ± 0.02 ^a^	20.25 ± 0.09 ^a^
SP2	102.01 ± 1.02 ^c^	32.97 ± 0.83 ^d^	57.71 ± 0.82 ^c^	30.23 ± 0.39 ^d^	54.65 ± 0.29 ^d^	54.77 ± 0.34 ^d^	3.00 ± 0.02 ^b^	8.87 ± 0.11 ^d^
SP3	154.89 ± 0.69 ^a^	130.47 ± 0.56 ^a^	133.25 ± 0.56 ^a^	83.90 ± 0.26 ^a^	95.51 ± 0.20 ^a^	86.04 ± 0.23 ^a^	2.87 ± 0.01 ^c^	13.09 ± 0.07 ^b^
SP4	73.42 ± 0.70 ^d^	56.18 ± 0.57 ^c^	58.41 ± 0.57 ^c^	73.34 ± 0.27 ^b^	92.62 ± 0.20 ^c^	77.58 ± 0.23 ^b^	1.81 ± 0.02 ^d^	11.07 ± 0.08 ^c^
DMP								
SP1	94.19 ± 1.11 ^d^	30.98 ± 0.95 ^d^	56.99 ± 0.97 ^d^	33.67 ± 0.31 ^d^	58.28 ± 0.25 ^c^	58.05 ± 0.27 ^d^	2.42 ± 0.02 ^b^	8.85 ± 0.10 ^d^
SP2	141.38 ± 0.65 ^b^	127.89 ± 0.55 ^b^	130.58 ± 0.56 ^b^	89.71 ± 0.18 ^a^	95.85 ± 0.14 ^a^	92.16 ± 0.16 ^a^	2.03 ± 0.01 ^d^	12.09 ± 0.06 ^c^
SP3	222.11 ± 0.84 ^a^	158.87 ± 0.71 ^a^	167.95 ± 0.73 ^a^	72.49 ± 0.24 ^c^	92.96 ± 0.19 ^b^	76.60 ± 0.21 ^c^	4.11 ± 0.01 ^a^	19.00 ± 0.08 ^a^
SP4	106.59 ± 0.68 ^c^	80.46 ± 0.58 ^c^	82.65 ± 0.59 ^c^	75.18 ± 0.19 ^b^	95.47 ± 0.15 ^a^	77.51 ± 0.17 ^b^	2.17 ± 0.01 ^c^	18.00 ± 0.06 ^b^
AMP								
SP1	181.94 ± 0.78 ^b^	153.55 ± 0.65 ^a^	165.16 ± 0.65 ^a^	84.22 ± 0.26 ^a^	91.57 ± 0.23 ^b^	90.89 ± 0.22 ^a^	2.52 ± 0.02 ^b^	13.37 ± 0.10 ^c^
SP2	119.10 ± 0.71 ^c^	94.18 ± 0.59 ^c^	96.12 ± 0.59 ^c^	78.61 ± 0.23 ^b^	96.25 ± 0.20 ^a^	80.50 ± 0.20 ^b^	2.15 ± 0.01 ^c^	18.18 ± 0.09 ^b^
SP3	101.35 ± 1.32 ^d^	30.81 ± 1.09 ^d^	61.85 ± 1.09 ^d^	30.50 ± 0.43 ^d^	52.96 ± 0.38 ^c^	57.76 ± 0.37 ^d^	2.46 ± 0.03 ^b^	9.67 ± 0.17 ^d^
SP4	222.59 ± 1.03 ^a^	133.84 ± 0.82 ^b^	144.42 ± 0.85 ^b^	60.62 ± 0.34 ^c^	91.09 ± 0.30 ^b^	65.50 ± 0.29 ^c^	4.20 ± 0.02 ^a^	22.58 ± 0.13 ^a^

PMP: pre-mating period, DMP: during mating period, AMP: after mating period, SEM: standard error of mean, VCL: curvilinear velocity, VSL: straight-line velocity, VAP: average path velocity, LIN: linearity of forward progression, STR: straightness, WOB: wobble, ALH: amplitude of lateral head displacement, BCF: beat-cross frequency. ^a–d^ Different letters within columns indicate differences between ejaculate subpopulations according to the different times during mating period. *p* < 0.05.

## Data Availability

The data presented in this study are available within the article.
